# Association of cardiovascular structure and function with cerebrovascular changes and cognitive function in older patients with end-stage renal disease

**DOI:** 10.18632/aging.102696

**Published:** 2020-01-07

**Authors:** Laurien E. Zijlstra, Stella Trompet, J. Wouter Jukema, Lucia J. M. Kroft, Jeroen de Bresser, Matthias J. P. van Osch, Sebastiaan Hammer, Marie-Noëlle Witjes, Marjolijn van Buren, Simon P. Mooijaart

**Affiliations:** 1 Department of Cardiology, Leiden University Medical Center, Leiden, The Netherlands; 2 Department of Gerontology and Geriatrics, Leiden University Medical Center, Leiden, The Netherlands; 3 Department of Radiology, Leiden University Medical Center, Leiden, The Netherlands; 4 Department of Radiology, HAGA Hospital, The Hague, The Netherlands; 5 Department of Nephrology, Leiden University Medical Center, Leiden, The Netherlands; 6 Department of Nephrology, HAGA Hospital, The Hague, The Netherlands

**Keywords:** arterial stiffness, cerebrovascular changes, dementia, end stage renal disease, systolic heart function

## Abstract

The Dutch prospective multicenter cohort study COPE (Cognitive decline in Older Patients with End stage renal disease) aimed to investigate the association of cardiovascular structure and function with cerebrovascular changes and cognitive function in 85 older patients with chronic kidney disease stage 4 and 5, awaiting either dialysis or conservative care. MRI was performed measuring aortic stiffness (pulse wave velocity [PWV]) and cardiac systolic function (ejection fraction and cardiac index). Outcomes were MRI-derived cerebrovascular changes (microbleeds, lacunes and white matter hyperintensities) and cognitive function (memory, executive function and psychomotor speed). Mean age was 76 years and 66% were male. No statistically significant associations were observed between cardiovascular parameters and cerebrovascular changes. Cognitive function was worse in patients with high compared to low PWV in all three cognitive domains. Although there were clinically relevant associations of high PWV with poor cognition in all domains, after adjustment for age, sex and education only the Trail Making Test A remained statistically significant (p=0.030). In conclusion, this study suggests that a higher PWV might be associated with lower cognitive function, suggesting that arterial stiffness may be an underlying mechanism of development of cognitive impairment in older patients with ESRD. Larger studies should replicate and extend these findings.

## INTRODUCTION

Cardiovascular diseases and cognitive impairment are frequent and increasingly prevalent, especially in older patients and patients with end-stage renal disease (ESRD) [1–4]. Both chronic kidney disease, especially ESRD, and cardiovascular diseases have been identified as independent risk factors for the development of microvascular damage and cerebral small vessel disease, which can lead to structural cerebrovascular changes and cognitive impairment [5–9]. It is, however, unknown how cardiovascular structure and function associates with brain structure and function in older patients with ESRD.

In ESRD nephrogenic factors as uremic toxins, anaemia and inflammation, are potential underlying mechanisms for the development of cerebrovascular changes and cognitive impairment [9]. Furthermore, cardiovascular risk factors can lead to microvascular damage in both the brain and kidney [9, 10]. The association between cardiovascular structure and function with both cerebrovascular changes and cognitive impairment can be divided into two possible mechanisms in the general population, namely increased arterial stiffness and impaired systolic heart function. Arterial stiffness can cause microvascular damage in the brain due to an increased impact of pulsatility on the microvasculature, which possibly alters brain structure or cognitive functioning [11–13]. Furthermore, impaired systolic heart function could cause cerebral ischemia because of hypoperfusion in the brain due to decreased cardiac output [14–17]. To what extent an altered cardiac structure and function play a role in cerebrovascular changes and cognitive impairment in older patients with ESRD remains unclear.

**Figure 1:**
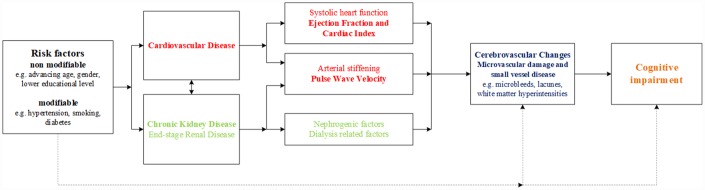
**The heart-kidney-brain axis.** Hypothesis of the current study and the potential underlying pathophysiological mechanisms in the heart-kidney-brain axis.

Figure 1 shows the hypothesis of the current study as well as the potential underlying mechanisms. The aim of this study was to investigate the association of cardiovascular structure and function with cerebrovascular changes and cognitive function in older patients with ESRD.

## RESULTS

**Figure 2:**
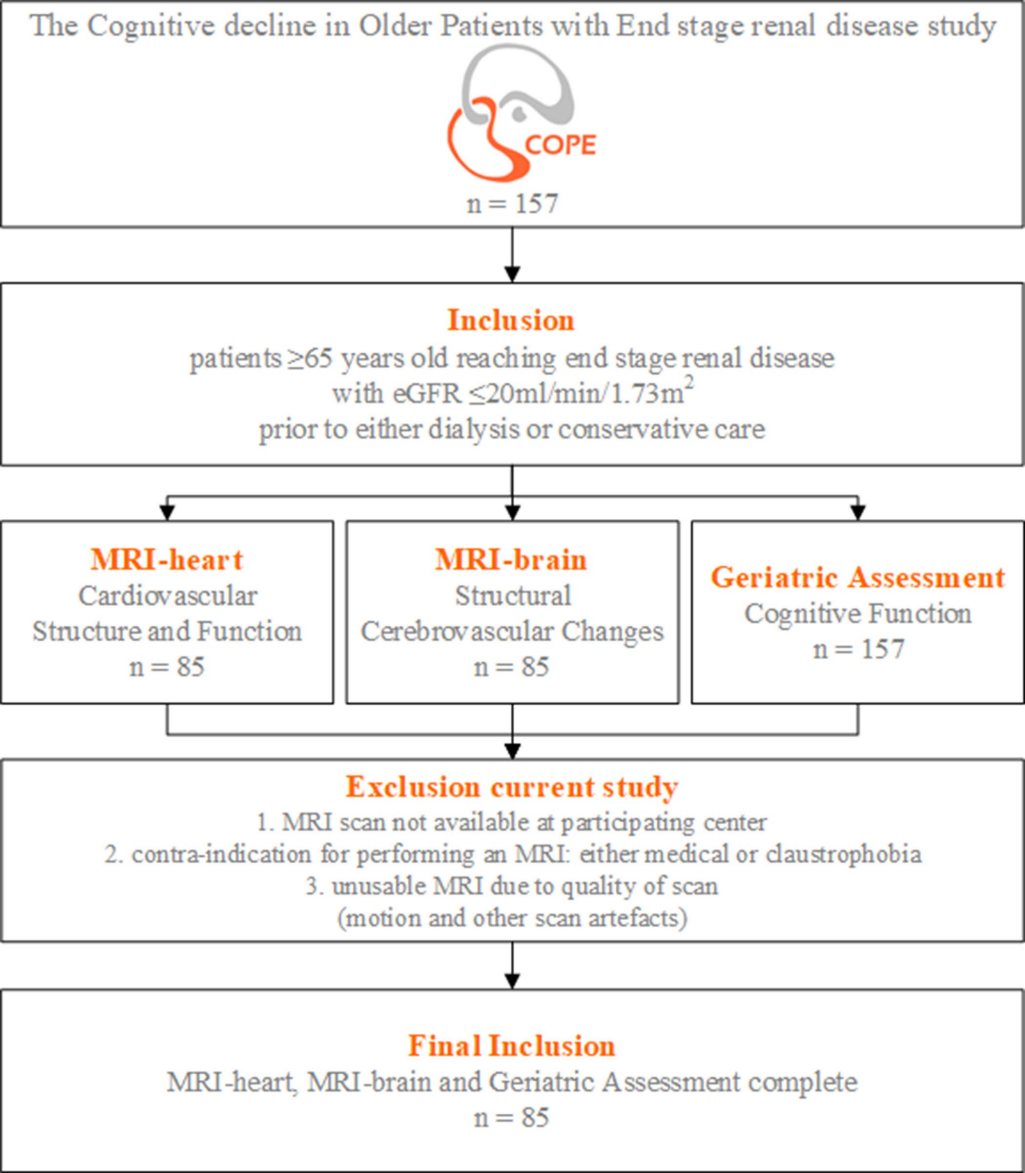
**Flowchart study population.** Inclusion and exclusion criteria of the COPE (The Cognitive decline in Older Patients with End stage renal disease) study.

Of the 157 patients included in the ‘The Cognitive decline in Older Patients with End stage renal disease’ (COPE) study, cardiac magnetic resonance imaging (MRI) scans were available for 85 participants, see the flowchart in Figure 2. Baseline characteristics of all patients are shown in Table 1. Mean±standard deviation (SD) of age was 75.6±6.9years and 56 (66%) patients were male. Mean±SD eGFR at time of inclusion was 15.8±4.2ml/min/1.73m^2^. The origin of primary kidney disease was non-vascular in 36% and vascular (mainly diabetes and hypertension) in 64% of all patients. Median [interquartile range (IQR)] pulse wave velocity (PWV) was 9.6m/s [7.8-13.0], ejection fraction (EF) 62% [51–66] and cardiac index (CI) 2.5l/min/m^2^ [2.1-3.0]. Global cognition in the total population was not impaired, measured by the MMSE with median [IQR] 28 [27–30] out of 30 points and also clock drawing with a median [IQR] of 12 [11–13]. Differences in baseline characteristics for each subgroup of PWV, EF and CI are shown in Supplementary Tables 1–3.

**Table 1:** Baseline characteristics total population (n=85).

Male gender, n (%)	56 (65.9)
Age, years; mean ± SD	75.6 ± 6.9
Race, Caucasian, n (%)	75 (88.2)
Higher educational level, n (%)	32 (37.6)
Primary kidney disease, n (%)	
Non-vascular cause	30 (35.7)
Vascular cause	54 (64.3)
*Comorbidity, n (%)*	
Diabetes mellitus	32 (37.6)
Peripheral vascular disease	16 (18.8)
Cerebral vascular accident	23 (27.1)
Heart failure	7 (8.2)
Coronary heart disease	18 (21.2)
Atrial fibrillation	17 (20.5)
Alcohol consumption, n (%)	45 (52.9)
Current smoking, n (%)	14 (16.5)
History of smoking, n (%)	49 (57.6)
*Medication use, n (%)*	
Polypharmacy (the use of ≥5 medications)	75 (88.2)
Antihypertensive medication	79 (92.9)
Beta-blockers	44 (51.8)
Diuretics	50 (58.8)
*Objective measures, mean ± SD*	
Blood pressure (mmHg)	
Systolic	150.3 ± 22.2
Diastolic	81.6 ± 11.8
eGFR (ml/min/1.73m^2^)	15.8 ± 4.2
Urea (mg/dL)	21.3 ± 6.3
Phosphate (mmol/L)	1.32 ± 0.29
Albuminuria (mg/24 hours)	771 ± 882
Troponin (ng/L)	0.052 ± 0.070
NT-proBNP (ng/L)	879 ± 1208
*Cardiovascular function, measured by MRI, median [IQR]*	
Pulse wave velocity (m/s)	9.6 [7.8-13.0]
Ejection fraction (%)	62 [51–66]
Cardiac index (l/min/m^2^)	2.5 [2.1-3.0]
*Cerebrovascular changes, measured by MRI, n (%) or mean ± SD*	
Presence of microbleeds	
Non-lobar	18 (21.2)
Lobar	32 (37.6)
Presence of lacunes*	39 (45.9)
Total white matter hyperintensities	16.1 ± 8.0
*Cognitive function performance, mean ± SD or median [IQR]*	
Global cognition	
Mini-Mental State Examination (points)	28 [27–30]
Visuoconstruction	
Clock drawing	12 [11–13]
Memory	
15-WVLT immediate recall	32.3 ± 10.1
15-WVLT delayed recall	6.3 ± 3.0
Visual Association Test	12 [11–12]
Executive function	
TMT-B (sec)	157.0 ± 72.5
SCWT III (sec)	166.0 ± 90.0
SCWT III corrected for SCWT II (sec)	84.0 ± 81.6
Psychomotor Speed	
LDST (correct in 60 sec)	22.9 ± 7.1
TMT-A (sec)	62.0 ± 39.0
SCWT II (sec)	82.5 ± 33.7

*Lacunes; both gliotic and hemorrhagic parenchymal defects subcortical, in brain stem and basal ganglia. Abbreviations: 15-WVLT, 15-Word Verbal Learning Test; eGFR, estimated glomerular filtration rate; LDST, Letter Digit Substitution Test; NT-proBNP, N-terminal pro b-type natriuretic peptide; SD, standard deviation; SCWT, Stroop Color and Word Test; TMT, Trail Making Test.

### Cerebrovascular changes

Table 2 shows the association between cardiac parameters and cerebrovascular changes. No statistically significant associations were observed between cardiac parameters and cerebrovascular changes. Patients with a high PWV, and therefore high aortic stiffness, more often than with low PWV, had more structural cerebrovascular changes, including more microbleeds (both non-lobar and lobar) and lacunes, and a higher mean total white matter hyperintensities (WMH), although differences were of unknown clinical relevance and not statistically significant. Similar non-significant results were seen for patients with a low compared to high EF and a low compared to high CI. A sensitivity analysis based on median PWV, EF and CI and a sensitivity analysis excluding patients with a history of CVA yielded similar results.

**Table 2:** Association of cardiovascular function parameters and cerebrovascular changes.

	**Better cardiovascular function**	**Worse cardiovascular function**	
	**Pulse wave velocity ≤10m/s** n = 45	**Pulse wave velocity >10m/s** n = 38	**p-value**	
			**Crude**	**Adjusted**
Presence of microbleeds, %				
Non-lobar	7 (16.7)	11 (28.9)	0.189	0.575
Lobar	13 (31.0)	18 (47.4)	0.132	0.105
Presence of lacunes, %	18 (42.9)	19 (50.0)	0.522	0.616
Total WMH, mean ± SE	15.5 ± 1.2	16.6 ± 1.4	0.561	0.438
	**Ejection fraction ≥50%** n = 65	**Ejection fraction <50%** n = 19		
Presence of microbleeds, %				
Non-lobar	13 (20.6)	5 (27.8)	0.520	0.563
Lobar	22 (34.9)	10 (55.6)	0.114	0.121
Presence of lacunes, %	29 (46.0)	10 (55.6)	0.476	0.767
Total WMH, mean ± SE	15.8 ± 0.9	16.9 ± 2.4	0.609	0.778
	**Cardiac index >2.2l/min/m^2^** n = 56	**Cardiac index ≤2.2l/min/m^2^** n = 27		
Presence of microbleeds, %				
Non-lobar	13 (23.6)	5 (20.0)	0.718	0.457
Lobar	23 (41.8)	8 (32.0)	0.403	0.382
Presence of lacunes, %	28 (50.9)	10 (40.0)	0.365	0.443
Total WMH, mean ± SE	17.0 ± 1.2	14.2 ± 1.3	0.151	0.166

P-values are assessed using linear or logistic regression models, unadjusted and adjusted for age and sex, comparing low versus high pulse wave velocity, ejection fraction and cardiac index.

Lacunes include both gliotic and hemorrhagic parenchymal defects subcortical, in brain stem and basal ganglia.

Abbreviations: SE, standard error; WMH, white matter hyperintensities.

### Cognitive function

Table 3 shows the association between cardiac parameters and cognitive function in three different domains, namely memory, executive function and psychomotor speed. The scores of all cognitive function tests were worse for patients with a high compared to low PWV in all three domains, statistical significance was reached for the Trail Making Test B (TMTB) with a difference of 39 seconds (p=0.009), for Trail Making Test A (TMTA) with a difference of 23 seconds (p=0.008) and for the Letter Digit Substitution Test (LDST) with a difference of 4 correct answers in 60 seconds (p=0.021). After adjustment for age, sex and education, only the TMTA, measuring psychomotor speed, remained statistically significant (p=0.030). No clinically relevant nor statistically significant associations were found in cognitive function comparing patients with low compared to high EF. Patients with a low compared to high CI had a worse memory function (both immediate and delayed recall), which remained statistically significant for delayed recall after adjustment (p=0.003). No statistically significant differences were found in executive function or psychomotor speed. A sensitivity analysis based on median PWV, EF and CI and a sensitivity analysis excluding patients with a history of CVA yielded similar results.

**Table 3:** Association of cardiovascular function parameters and cognitive function.

	**Better Cardiovascular function**	**Worse Cardiovascular function**		
	**PWV ≤10m/s** n=45	**PWV >10m/s** n=38	** p-value**	
			**Crude**	**Adjusted**
**Memory**				
15-Word Verbal Learning Test immediate recall ↓	33.3 ± 1.2	31.6 ± 2.0	0.455	0.899
15-Word Verbal Learning Test delayed recall ↓	6.6 ± 0.4	5.9 ± 0.6	0.307	0.719
**Executive function**				
Trail Making Test B (sec) ↑	140.6 ± 9.6	179.3 ± 12.2	0.009	0.184
SCWT III (sec) ↑	158.3 ± 15.6	173.5 ± 11.8	0.439	0.339
SCWT III corrected for SCWT II (sec) ↑	80.5 ± 14.0	87.6 ± 11.1	0.692	0.148
**Psychomotor speed**				
Letter Digit Substitution Test (correct in 60 sec) ↓	24.6 ± 0.9	20.9 ± 1.3	0.021	0.179
Trail Making Test A (sec) ↑	51.6 ± 2.5	74.6 ± 8.7	0.008	0.030
Stroop Color and Word Test II (sec) ↑	77.8 ± 3.9	87.0 ± 6.7	0.241	0.361
	**EF ≥50%** n=65	**EF <50%** n=19		
**Memory**				
15-Word Verbal Learning Test immediate recall ↓	32.1 ± 1.2	32.5 ± 2.8	0.881	0.621
15-Word Verbal Learning Test delayed recall ↓	6.4 ± 0.4	5.7 ± 0.8	0.426	0.664
**Executive function**				
Trail Making Test B (sec) ↑	160.4 ± 8.9	153.4 ± 19.2	0.809	0.728
SCWT III (sec) ↑	166.7 ± 11.7	164.8 ± 19.1	0.940	0.669
SCWT III corrected for SCWT II (sec) ↑	86.4 ± 10.3	75.9 ± 19.8	0.633	0.392
**Psychomotor Speed**				
Letter Digit Substitution Test (correct in 60 sec) ↓	23.4 ± 0.8	21.3 ± 1.9	0.265	0.383
Trail Making Test A (sec) ↑	59.2 ± 4.4	73.4 ± 12.3	0.186	0.274
Stroop Color and Word Test II (sec) ↑	80.9 ± 3.2	88.9 ± 12.8	0.377	0.459
	**CI >2.2 l/min/m^2^** n=56	**CI≤2.2 l/min/m^2^** n=27		
**Memory**				
15-Word Verbal Learning Test immediate recall ↓	33.2 ± 1.4	30.1 ± 1.9	0.207	0.219
15-Word Verbal Learning Test delayed recall ↓	6.9 ± 0.4	4.9 ± 0.5	0.004	0.003
**Executive function**				
Trail Making Test B (sec) ↑	149.6 ± 8.7	176. ± 16.6	0.156	0.191
SCWT III (sec) ↑	164.5 ± 13.1	171.4 ± 15.6	0.748	0.978
SCWT III corrected for SCWT II (sec) ↑	85.1 ± 12.9	84.3 ± 10.0	0.966	0.712
**Psychomotor Speed**				
Letter Digit Substitution Test (correct in 60 sec) ↓	23.4 ± 0.9	21.5 ± 1.5	0.266	0.349
Trail Making Test A (sec) ↑	59.9 ± 4.4	68.1 ± 10.1	0.384	0.414
Stroop Color and Word Test II (sec) ↑	80.2 ± 4.6	87.1 ± 6.5	0.387	0.428

Values are mean ± SE. ↓↑ indicates that a higher (↑) or lower (↓) score means a worse cognitive function.

P-values are assessed using linear regression models, unadjusted and multivariate adjusted for age, sex and education, comparing low versus high pulse wave velocity, ejection fraction and cardiac index.

Abbreviations: CI, cardiac index; EF, ejection fraction; PWV, pulse wave velocity

## DISCUSSION

The main findings of this explorative study are as follows. First, higher PWV associated with all measures of cognitive impairment, albeit this was only statistically significant for the association of PWV with the TMTA, measuring psychomotor speed. Second, no statistically significant differences in the association between cardiovascular structure and function and structural cerebrovascular changes were found.

Although the association of arterial stiffness and brain pathology has been described previously in patients with ESRD, studies are limited, and included in general only cerebrovascular changes [18] or cognition [19], and in case of the latter limited tests for global cognition, instead of differentiating between various functional domains [20, 21]. In the general population, the influence of arterial stiffness on both cerebrovascular changes and cognitive function has been more extensively studied. Cardiovascular risk factors such as hypertension result in arterial stiffening that can be measured in the aorta as increased PWV, an important and independent determinant of arterial disease [11, 12]. Due to an impaired Windkessel effect aortic stiffness might increase the impact of cardiac pulsations on the cerebral microvasculature leading to cerebral small vessel diseases and cognitive impairment [13], as was recently confirmed by a systematic review [22]. Studies have shown independent associations of impaired systolic heart function on cerebrovascular changes or cognitive function, probably due to cerebral ischemia because of hypoperfusion in the brain due to decreased cardiac output [14–17], including in patients with ESRD [23]. However, it might have a more multifactorial dependency, like for instance whether patients clinically have heart failure. Previous studies have shown associations of heart failure [14], and also associations of both EF and CI in patients with heart failure [15], with cerebrovascular changes and cognitive impairment. Furthermore, treatment of heart failure, with for example cardiac resynchronization therapy or heart transplantation, can improve cognitive function, partly due to improvement of cerebral blood flow [24–26]. Taken together, our findings that arterial stiffness may be an underlying mechanism of development of cognitive impairment is in line with known literature in both the general population, but also in patients with ESRD.

We investigated two hypotheses as underlying pathophysiological mechanisms in the heart-kidney-brain axis, namely arterial stiffness and systolic heart function (Figure 1). Few associations reached statistical significance, possibly due to the relatively low number of participants in the study. However, for PWV, all 4 cerebrovascular changed parameters, and all 8 cognitive associates point in the same direction. The magnitudes of association in most cognitive parameters were well above what could be considered clinically relevant, which is unlikely the result of chance. Therefore, we conclude that our results suggest that PWV is a potential predictor of cognitive function in older patients with ESRD. These results, however, should be considered as "suggestive" and need replication in larger cohorts. The contributing role of systolic cardiac function on cerebrovascular changes and cognitive impairment seemed limited in our COPE population, possibly due to a low percentage of patients with manifest symptoms of clinical heart failure (7%). In addition, although EF and CI are both parameters of systolic cardiac function, values within the same patients are not always concordant. In our population, patients with a low EF had a relatively normal mean CI and vice versa. Cardiac output has been pointed out previously to be a superior reflection of systemic blood flow and cerebral blood flow than EF, especially in patients without heart failure [27]. It might explain that low CI was a better predictor of cognitive impairment (memory domain) than low EF in our population.

### Strengths and limitations

The COPE study is a unique observational study in older patients with ESRD with a mean eGFR of 16ml/min/1.73m^2^, prior to either dialysis or conservative care, with comprehensive measurements of cardiovascular function, cerebrovascular changes and cognitive function.

However, some limitations should be mentioned. Patient numbers were relatively limited, and although cardiovascular comorbidity was common, patients had a relatively normal cardiac function, with only 19 patients available with EF ≤50% and 27 patients with CI <2.2l/min/m^2^, limiting the power to find an association between cardiovascular function and cerebrovascular changes or cognitive function of a smaller magnitude. This limited us to merely observe possible trends suitable for future research.

## CONCLUSIONS

In conclusion, this exploratory study suggests that a higher PWV is associated with lower cognitive function, but not with increased cerebrovascular changes in older patients with ESRD, suggesting that arterial stiffness may be an underlying mechanism of development of cognitive impairment.

Larger studies should replicate and extend on these findings, as identifying the mechanisms involved in cerebrovascular changes and cognitive impairment can be the first step towards prevention strategies. Prevention is of utmost importance, as the Framingham Heart Study have showed that earlier diagnosis and effective treatment of risk factors or proven vascular disease, can possibly lead to a decline in incidence of dementia [28]. Furthermore, future research should also focus on other potential biomarkers as miRNAs or metabolomics to unravel specific pathophysiological mechanisms in this interaction between the heart, kidney and brain and thereafter on potential interventions to prevent cerebrovascular changes and cognitive impairment.

## MATERIALS AND METHODS

Data of ‘The Cognitive decline in Older Patients with End stage renal disease’ (COPE) study were used, a Dutch prospective, multicenter cohort study. A detailed description of the rationale and design of COPE, including all in- and exclusion criteria, has been published previously [29]. In summary, patients ≥65 years old reaching ESRD with eGFR ≤20ml/min/1.73m^2^ (CKD stage 4 and 5) were included, prior to either dialysis or conservative care. The main study objective was to study the association between underlying pathophysiological mechanisms and cognitive decline in patients with ESRD. For this purpose, magnetic resonance imaging (MRI) of the heart and brain and extensive neurocognitive testing were performed. For the current analysis patients without a cardiac MRI were excluded, see flowchart in Figure 2. Written informed consent was obtained from all study participants. The study protocol was approved by the medical ethics committees (METC) of all participating centers (Leiden University Medical Center [LUMC, Leiden], HAGA Hospital [Den Haag], Dialysis Center Zoetermeer [Zoetermeer], Reinier de Graaf Group [Delft] and Jeroen Bosch Hospital [Den Bosch]).

### Magnetic resonance imaging

All MRI scans were made on a 3T Philips Achieva MRI scanner (Philips, Best, The Netherlands). Brain MRI was made with a 32 channel receive coil, heart MRI with an 8-channel receive coil.

### Cardiovascular structure and function

The cardiac MRI protocol included flow sensitive imaging by phase contrast MRI for pulse wave velocity (PWV), measuring aortic stiffness [29]. Furthermore, the protocol included TFE (turbo field echo) multi-slice multi-phase cine-imaging of the left ventricle for systolic function, including ejection fraction (EF) and cardiac index (CI). Ejection fraction is the percentage of blood ejected out of the ventricles with each contraction (stroke volume divided by the end diastolic volume in %). Cardiac index is calculated as cardiac output (stroke volume multiplied by heart rate) and corrected for body surface area with use of the Du Bois formula (in l/min/m^2^) [30]. After exclusion of scans with poor quality (artifacts mainly caused by movement or distortions) PWV was available for 83 patients, EF for 84 patients and CI for 83 patients.

### Cerebrovascular changes

The brain MRI protocol included 3D FLAIR (fluid attenuated inversion recovery) and T2-weighted brain MRI images, which were scored for the presence of markers of small vessel disease, including white matter hyperintensities (WMH) and lacunes. Susceptibility weighted imaging was used to score the presence and distribution of cerebral microbleeds. Cerebrovascular changes were rated as presence of (non) lobar microbleeds, presence of lacunes and grade of WMH according to the Scheltens score [31]. For three patients the brain MRI was of insufficient quality for the rating, due to motion artefacts.

### Cognitive function

Detailed description of the comprehensive geriatric assessment and neuropsychological tests used in COPE has been published previously [29]. Outcome variables were derived from seven widely used neuropsychological tests in five different cognitive domains. First, global cognition was measured by the Mini-Mental State Examination (MMSE), with a general cut-off point of 24 out of 30 points [32]. Visuoconstructible abilities were assessed using the clock drawing test, which is often used as dementia screening test, with scores ranging from 0 to 14 points based on accuracy [33, 34]. Memory was assessed using two tests, namely the 15-Word Verbal Learning Test (15-WVLT), which measures immediate recall (total outcome of five presentations) and delayed recall after 20 minutes [35–37]. Memory was also assessed using the Visual Association Test (VAT) of which the score is based on the number of completed associations reported in two trials [38]. Executive functioning was assessed using the Trail Making Test B (TMTB), which is a switching task. The score of the TMTB is the number of seconds required to complete the task [39, 40]. Furthermore, the Stroop Color and Word Test (SCWT) card III (interference card) was used [41–43]. The SCWT consists of three parts, namely reading color names (card I), naming colored patches (card II) and naming color names printed in incongruously colored ink (card III). The time in seconds required to read the names or to identify colors is recorded. We used an abbreviated version of the test with 40 elements [44]. Psychomotor speed was assessed with the Letter Digit Substitution Test (LDST), Trail Making Test A (TMTA) and SCWT card II (naming colored patches). The LDST is a modification of the procedurally identical Symbol-Digits Modalities Test, with an outcome variable of total number of correct entries completed in 60 seconds [45, 46]. The score of the TMTA, testing visual attention, is the counted the same as the TMTB [39, 40].

### Statistical analysis

Cardiac parameters are dichotomized by clinical cut-off values based on current guidelines [47, 48]. These cut-off values are ≤10m/s and >10m/s for PWV, respectively no aortic stiffness versus aortic stiffness, ≤50% and >50% for EF, and <2.2l/min/m^2^ and ≥2.2L/min/m^2^ for CI, both respectively low versus high. All categorical data are presented as numbers with percentages. All continuous data are presented as mean ± standard error or ± standard deviation, or median with interquartile range. Baseline differences between cardiac parameters are assessed using an independent t-test, Mann-Whitney U test or chi-square test. Multivariate linear or logistic regression models are used to assess the associations of cardiac function with cerebrovascular changes and cognitive function. All analyses were adjusted for prespecified confounders, namely age and gender, and also education in case of cognitive function analyses. The data were analyzed using IBM SPSS Statistics version 23. P-values lower than 0.05 were considered statistically significant.

## Supplementary Material

Supplementary Tables
