# Supplementary material for: Association of cardiovascular structure and function with cerebrovascular changes and cognitive function in older patients with end-stage renal disease
Source: Aging (Albany NY). 2020 Jan 7;12(2):1496–511. doi: 10.18632/aging.102696 (PMC7053617; doi:10.18632/aging.102696)
Supplement: Supplementary Tables [file aging-12-102696-s001..pdf]

SUPPLEMENTARY TABLES

Supplementary Table 1. Determinants of pulse-wave velocity.

	Better cardiovascular function	Worse cardiovascular function	p-value
	Pulse wave velocity ≤10 m/s<br>n = 45	Pulse wave velocity >10 m/s<br>n = 38	
Pulse wave velocity (m/s), mean ± SD	7.8 ± 1.4	14.1 ± 3.8	
Male gender, n (%)	33 (73.3)	22 (57.9)	0.138
Age, years; mean ± SD	73.0 ± 6.4	78.4 ± 6.4	<0.0001
Race, Caucasian, n (%)	40 (88.9)	34 (89.5)	0.696
Higher educational level, n (%)	15 (33.3)	15 (39.5)	0.562
Primary kidney disease, n (%)			0.652
Non-vascular cause	16 (35.6)	14 (36.8)	
Vascular cause	28 (62.2)	24 (63.2)	
Comorbidity, n (%)			
Diabetes mellitus	17 (37.8)	15 (39.5)	0.874
Peripheral vascular disease	8 (17.8)	7 (18.4)	0.940
Cerebral vascular accident	13 (28.9)	10 (26.3)	0.794
Heart failure	3 (6.7)	3 (7.9)	0.830
Coronary heart disease	12 (26.7)	8 (21.1)	0.551
Atrial fibrillation	8 (17.8)	9 (23.7)	0.506
Alcohol consumption, n (%)	27 (61.4)	17 (44.7)	0.132
Current smoking, n (%)	10 (22.7)	4 (10.5)	0.143
History of smoking, n (%)	26 (76.5)	22 (66.7)	0.373
Objective measures, mean ± SD			
eGFR (ml/min/1.73m^2)	16.2 ± 4.5	15.4 ± 3.8	0.393
Urea (mg/dL)	20.3 ± 5.6	22.5 ± 7.1	0.134
Phosphate (mmol/L)	1.31 ± 0.30	1.33 ± 0.28	0.800
Albuminuria (mg/24 hours)	815 ± 900	712 ± 874	0.683
Troponin (ng/L)	0.056 ± 0.083	0.048 ± 0.053	0.715
NT-proBNP (ng/L)	1054 ± 1386	707 ± 985	0.691
Blood pressure (mmHg)			
Systolic	149.2 ±22.9	151.9 ± 21.9	0.601
Diastolic	82.8 ± 10.2	80.2 ± 13.7	0.342
Ejection fraction (%)	58.5 ± 10.9	59.4 ± 9.8	0.686
Cardiac index (l/min/m^2)	2.6 ± 0.6	2.6 ± 0.4	0.652

P-values are assessed using an independent t-test, Mann-Whitney U test or chi-square test. Abbreviations: eGFR, estimated glomerular filtration rate; NT-proBNP, N-terminal pro b-type natriuretic peptide; SD, standard deviation;

Supplementary Table 2. Determinants of ejection fraction.

	Better cardiovascular function	Worse cardiovascular function	p-value
	Ejection fraction >50% n = 65	Ejection fraction <50% n = 19	
Ejection fraction (%), mean ± SD	63.2 ± 6.2	42.3 ± 7.3	
Male gender, n (%)	40 (61.5)	15 (78.9)	0.160
Age, years; mean ± SD	75.4 ± 6.8	76.6 ± 7.5	0.471
Race, Caucasian, n (%)	55 (84.6)	19 (100.0)	0.506
Higher educational level, n (%)	21 (32.3)	10 (52.6)	0.106
Primary kidney disease, n (%)			0.860
Non-vascular cause	23 (35.4)	7 (36.8)	
Vascular cause	41 (63.1)	12 (63.2)	
Comorbidity, n (%)			
Diabetes mellitus	27 (41.5)	5 (26.3)	0.229
Peripheral vascular disease	10 (15.4)	6 (31.6)	0.114
Cerebral vascular accident	14 (21.5)	8 (42.1)	0.073
Heart failure	3 (4.6)	4 (21.1)	0.023
Coronary heart disease	15 (23.1)	6 (31.6)	0.452
Atrial fibrillation	12 (18.5)	6 (31.6)	0.220
Alcohol consumption, n (%)	32 (50.0)	13 (68.4)	0.157
Current smoking, n (%)	3 (15.8)	11 (17.2)	0.886
History of smoking, n (%)	33 (63.5)	16 (100.0)	0.004
Objective measures, mean ± SD			
eGFR (ml/min/1.73m^2)	16.0 ± 4.4	14.9 ± 3.4	0.303
Urea (mg/dL)	21.2 ± 6.3	21.7 ± 6.7	0.763
Phosphate (mmol/L)	1.31 ± 0.30	1.40 ± 0.23	0.243
Albuminuria (mg/24 hours)	754 ± 849	893 ± 1101	0.674
Troponin (ng/L)	0.050 ± 0.077	0.060 ± 0.032	0.008
NT-proBNP (ng/L)	572 ± 915	2020 ± 1482	<0.0001
Blood pressure (mmHg)			
Systolic	151.3 ± 22.3	148.0 ± 22.3	0.588
Diastolic	80.1 ± 11.7	85.8 ± 12.1	0.103
Pulse wave velocity (m/s)	10.5 ± 4.1	11.3 ± 4.8	0.447
Cardiac index (l/min/m^2)	2.6 ± 0.7	2.5 ± 0.7	0.498

P-values are assessed using an independent t-test, Mann-Whitney U test or chi-square test. Abbreviations: eGFR, estimated glomerular filtration rate; NT-proBNP, N-terminal pro b-type natriuretic peptide; SD, standard deviation;

Supplementary Table 3. Determinants of cardiac Index.

	Better cardiovascular function<br>Cardiac index >2.2 l/min/m^2<br>n = 56	Worse cardiovascular function<br>Cardiac index ≤2.2 l/min/m^2<br>n = 27	p-value
Cardiac index (l/min/m^2), mean ± SD	2.9 ± 0.5	1.9 ± 0.3	
Male gender, n (%)	39 (69.6)	16 (59.3)	0.349
Age, years; mean ± SD	75.4 ± 6.4	76.2 ± 7.7	0.675
Race, Caucasian, n (%)	48 (85.7)	26 (96.3)	0.373
Higher educational level, n (%)	21 (37.5)	10 (37.0)	0.967
Primary kidney disease, n (%)			0.690
Non-vascular cause	19 (33.9)	10 (38.5)	
Vascular cause	37 (66.1)	16 (61.5)	
Comorbidity, n (%)			
Diabetes mellitus	23 (41.1)	9 (33.3)	0.497
Peripheral vascular disease	13 (23.2)	3 (11.1)	0.190
Cerebral vascular accident	16 (28.6)	7 (25.9)	0.801
Heart failure	3 (5.4)	4 (14.8)	0.146
Coronary heart disease	14 (25.0)	7 (25.9)	0.928
Atrial fibrillation	9 (16.1)	9 (33.3)	0.074
Alcohol consumption, n (%)	29 (52.7)	15 (55.6)	0.809
Current smoking, n (%)	8 (14.5)	5 (18.5)	0.643
History of smoking, n (%)	32 (69.6)	17 (77.3)	0.508
Objective measures, mean ± SD			
eGFR (ml/min/1.73m^2)	15.3 ± 4.0	17.0 ± 4.4	0.077
Urea (mg/dL)	21.4 ± 6.2	21.3 ± 6.8	0.972
Phosphate (mmol/L)	1.33 ± 0.31	1.32 ± 0.25	0.925
Albuminuria (mg/24 hours)	744 ± 754	836 ± 1160	0.737
Troponin (ng/L)	0.049 ± 0.048	0.038 ± 0.031	0.751
NT-proBNP (ng/L)	944 ± 1208	799 ± 1266	0.308
Blood pressure (mmHg)			
Systolic	149.5 ± 20.4	153.0 ± 26.6	0.516
Diastolic	80.0 ± 12.8	84.6 ± 9.0	0.110
Pulse wave velocity (m/s)	10.4 ± 3.9	11.2 ± 4.8	0.417
Ejection fraction (%)	59.6 ± 10.2	56.0 ± 12.5	0.168

P-values are assessed using an independent t-test, Mann-Whitney U test or chi-square test. Abbreviations: eGFR, estimated glomerular filtration rate; NT-proBNP, N-terminal pro b-type natriuretic peptide; SD, standard deviation;

Supplementary Table 4. univariate associations of blood pressure and pulse pressure with cerebrovascular changes values are beta ± SE.

	Systolic blood pressure		Diastolic blood pressure		Pulse Pressure	
	**beta**	**P-value**	**beta**	**P-value**	**beta**	**P-value**
Presence of microbleeds						
Non-lobar	0.021±0.01	0.100	0.000±0.02	0.999	0.023±0.01	0.085
Lobar	0.025±0.01	0.033	0.023±0.02	0.255	0.019±0.01	0.103
Presence of lacunar infarction	0.005±0.01	0.628	-0.007±0.02	0.701	0.008±0.01	0.468
Total WMH	0.024±0.04	0.559	-0.120±0.08	0.120	0.066±0.04	0.135

P-values are assessed using linear or logistic regression models. Lacunes include both gliotic and hemorrhagic parenchymal defects subcortical, in brain stem and basal ganglia. Abbreviations: WMH, white matter hyperintensities.

Supplementary Table 5. Univariate associations of blood pressure and pulse pressure with cognitive function values are beta ± SE.

	Systolic blood pressure		Diastolic blood pressure		Pulse pressure	
	**beta**	**P-value**	**beta**	**P-value**	**beta**	**P-value**
**Memory**						
15-WVLT immediate recall	-0.030±0.05	0.564	0.124±0.10	0.204	-0.071±0.05	0.189
15-WVLT delayed recall	0.003±0.02	0.842	0.044±0.03	0.139	-0.010±0.02	0.548
**Executive function**						
Trail Making Test B (sec)	1.134±0.33	0.001	0.312±0.68	0.645	1.143±0.35	0.002
Stroop Color Word Test III (sec)	0.575±0.45	0.207	-1.231±0.87	0.163	0.973±0.46	0.038
SCWT III corrected for SCWT II (sec)	0.271±0.43	0.527	-1.609±0.81	0.051	0.754±0.44	0.088
**Psychomotor speed**						
LDST (correct in 60 sec)	-0.066±0.03	0.058	-0.022±0.07	0.741	-0.066±0.04	0.072
Trail Making Test A (sec)	0.531±0.20	0.009	0.418±0.39	0.287	0.438±0.21	0.037
Stroop Color Word Test II (sec)	0.261±0.16	0.100	0.215±0.30	0.472	0.221±0.17	0.187

P-values are assessed using linear regression models. Abbreviations: 15-WVLT, 15-Word Verbal Learning Test; LDST, Letter Digit Substitution Test; SCWT, Stroop Color Word Test.
